# A healthcare worker's wedding during the COVID‐19 pandemic: Mental healthcare in the aftermath of an outbreak

**DOI:** 10.1002/pcn5.44

**Published:** 2022-09-13

**Authors:** Kyohei Otani, Atsumi Miura, Hiroyuki Miyai, Haruko Fukushima, Kunitaka Matsuishi

**Affiliations:** ^1^ Department of Psychiatry Kobe City Medical Center General Hospital, Chuo‐ku Kobe Hyogo Japan

In many countries, gatherings have been restricted where COVID‐19 infection rates have not subsided; weddings are subject to these restrictions.^1^ Although vaccinations have reduced the risk of COVID‐19, outbreaks at indoor events have occurred despite a high proportion of vaccinated attendees.^2^ These outbreaks have significantly harmed the mental health of healthcare workers.

We report on a frontline healthcare worker who had a wedding during the COVID‐19 pandemic and struggled. The “groom” was continually subjected to harassment at work during the spread of the infection, which forced him to take a leave of absence.

The groom was a hospital employee in his 20s who visited one of the authors with complaints of insomnia. With his bride, he had planned a wedding before the COVID‐19 pandemic. The wedding was postponed unavoidably due to the emergency declared in Japan at the beginning of the second wave.

The day before their wedding, the third wave began, and the hospital declared a period of extended infection and asked the staff to refrain from holding meetings. The hospital staff members were nervous to attend the wedding. Despite the infection‐control measures, they urged the wedding party not to have an after‐party. The newly married couple spent days feeling depressed after their wedding. One year later, when the vaccine rate rose in Japan, a small social gathering was organized. During the gathering, stories rehashed. The patient, the groom, missed work.

This case illustrates the difficulty of holding weddings among medical professionals during the ongoing pandemic. Cluster transmission of COVID‐19 at weddings has been observed worldwide^3^; weddings often involve clustering.^4^ In addition to the fear of infection and infecting family members, healthcare workers are fearful of bringing COVID‐19 into the workplace. Patients and employees needed to be quarantined following a cluster outbreak, which severely restricts hospital operations, therefore they are hesitant to organize weddings and feel exhausted attending them. Even without weddings, they are under tremendous stress.^5^ They suffer from anxiety and depression and face ethical dilemmas between their commitment to their patients and occupation, and their responsibility to their families^6^; they may also be stigmatized by their friends and community. They may be concerned about neglecting their COVID‐19 infection and facing criticism for their casual attitude towards it. Their fear of infecting themselves and guilt of possibly infecting others, or causing trouble, torment medical staff. Feelings of guilt can lead to morale decline and, as a moral injury, cause strong trauma to the staff who treat and care for patients and residents.^7^ During the COVID‐19 pandemic, not only were hospital staff unable to fulfill their duties, but they also faced various forms of discrimination. Additionally, the wedding took place right before the commencement of the third wave. Except for the bride and groom, who were still in their “honeymoon” phase, most medical professionals experienced the disillusionment phase as they faced the reality of COVID‐19 (Figure 1). Most medical practitioners may repeatedly find themselves in the disillusionment phase, tied to the rise and fall of COVID‐19 infection rates and repeated rebuilding phases.

**Figure 1:**
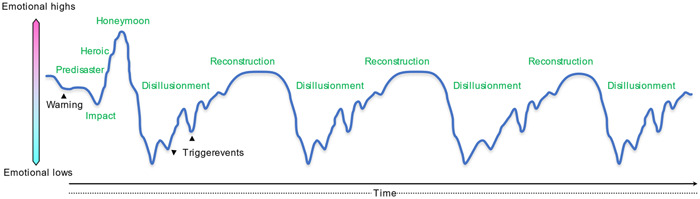
Model diagram of the general psychological changes in the affected population^10^ and the subsequent psychological changes associated with the infectious situation during the COVID‐19 pandemic

Another factor contributing to this is the unique mentality of the Japanese. While governments in Europe and the USA enforced lockdowns and banned gatherings during epidemics, Japan, being a self‐regulating country, did not need to do so. Japanese people are disposed to be attentive and sympathetic towards their surroundings, and traditionally have a sense of group cohesiveness or group consciousness that distinguishes between insiders and outsiders. This has propelled discrimination against those who have COVID‐19 or are suspected of spreading it.^8^

Therefore, we directed our mental healthcare team to listen to staff concerns in a multidisciplinary manner, and obtained data through e‐mail, phone calls, and other non‐face‐to‐face means. We also shared information on how the hospital had responded to issues related to COVID‐19, which can help eliminate concerns regarding unaddressed issues and increase the sense of security in the workplace. This was frequently requested in the free‐text summaries analyzed in our research.^9^

Two years after the outbreak, while infection anxiety is decreasing, the workload and exhaustion may be increasing. We hope that the sense of fighting together with COVID‐19 has been raised by the full supply of infection prevention equipment, allowance from the foundation (Grants for workers responding to COVID‐19 and Kobe medical staff support fund), and heartwarming assistance from citizens and companies.

The groom took sleeping pills which improved his sleep. He could manage his daily life at home and was physically active and reinstated. A wedding is a once‐in‐a‐lifetime event that should be celebrated without the shadow of mental health repercussions even when pandemic is ongoing.

## AUTHOR CONTRIBUTIONS

Kyohei Otani, Atsumi Miura, Hiroyuki Miyai, Haruko Fukushima, and Kunitaka Matsuishi were involved in study design. Kyohei Otani contributed to data analysis. Kyohei Otani, Haruko Fukushima, and Kunitaka Matsuishi contributed to the acquisition of data. Kyohei Otani drafted the initial manuscript. Haruko Fukushima and Kyohei Otani drafted the initial figure, which was then revised by Kyohei Otani. All authors approved the final manuscript.

## CONFLICT OF INTEREST

The authors declare no conflict of interest.

## ETHICS APPROVAL STATEMENT

We have obtained a release from the patient giving us permission to publish. The study was conducted in accordance with the Declaration of Helsinki.

## PATIENT CONSENT STATEMENT

Written consent from the patient was obtained.

## CLINICAL TRIAL REGISTRATION

N/A

## Data Availability

N/A
